# Exploring bacterial growth and recolonization after preoperative hand disinfection and surgery between operating room nurses and non-health care workers: a pilot study

**DOI:** 10.1186/s12879-018-3375-3

**Published:** 2018-09-17

**Authors:** Camilla Wistrand, Bo Söderquist, Karin Falk-Brynhildsen, Ulrica Nilsson

**Affiliations:** 1 Department of Cardiothoracic Surgery and Vascular Surgery, University Hospital in Örebro, Örebro, Sweden; 2 School of Health Sciences, Faculty of Medicine and Health, Örebro University, SE 701 82 Örebro, Sweden; 3 School of Medical Sciences, Faculty of Medicine and Health, Örebro University, SE 701 82 Örebro, Sweden; 4 Departments of Laboratory Medicine, Clinical Microbiology, and Infectious diseases, Örebro University Hospital, 701 85 Örebro, Sweden; 5 Division of Nursing,DepartmentofNeurobiology,Care Sciences,and Society,Karolinska Institute and Perioperative Medicine, Karolinska University Hospital, SE 14183 Stockholm, Sweden

**Keywords:** Bacterial growth, Hand disinfection, Preoperative, Cross infection, Bacterial re-colonization, Surgical gloves, Intraoperative, Surgery, Surgical site infections

## Abstract

**Background:**

To prevent cross infection the surgical team perform preoperative hand disinfection before dressed in surgical gowns and gloves. Preoperative hand disinfection does not make hands sterile and the surgical glove cuff end has been regarded as a weak link, since it is not a liquid-proof interface. The aims were to investigate if there were differences in bacterial growth and recolonization of hands between operating room nurses and non-health care workers as well as to investigate if bacterial growth existed at the surgical glove cuff end during surgery.

**Methods:**

This pilot project was conducted as an exploratory comparative clinical trial. Bacterial cultures were taken from the glove and gown interface and at three sites of the hands of 12 operating room nurses and 13 non-health care workers controls directly after preoperative hand disinfection and again after wearing surgical gloves and gowns. Colony forming units were analysed with Mann-Whitney U test and Wilcoxon Sign Ranks test comparing repeated measurements. Categorical variables were evaluated with chi-square test or Fisher’s exact test.

**Results:**

Operating room nurses compared to non-health care workers had significant higher bacterial growth at two of three culture sites after surgical hand disinfection. Both groups had higher recolonization at one of the three culture sites after wearing surgical gloves. There were no differences between the groups in total colony forming units, that is, all sampling sites. Five out of 12 of the operating room nurses had bacterial growth at the glove cuff end and of those, four had the same bacteria at the glove cuff end as found in the cultures from the hands. Bacteria isolated from the glove cuff were *P. acnes*, *S. warneri*, *S. epidermidis* and *Micrococcus* species, the CFU/mL ranged from 10 to 40.

**Conclusions:**

There were differences in bacterial growth and re-colonization between the groups but this was inconclusive. However, bacterial growth exists at the glove cuff and gown interface, further investigation in larger study is needed, to build on these promising, but preliminary, findings.

**Trial registration:**

Trial registration was performed prospectively at Research web (FOU in Sweden, 117,971) 14/01/2013, and retrospectively at ClinicalTrials.gov (NCT02359708). 01/27/2015.

## Background

Prevention of surgical site infections (SSIs) is important to avoid patient suffering and death and to lower the cost of health care providers [1]. Depending on where the SSI is located, an SSI can be devastating for the patient, as well as costly for society. For example, a severe SSI might be one that occurs after open heart surgery, where a deep sternal wound infection can double or even triple the usual cost of treatment [2–4]. In an intraoperative environment causative bacteria for SSI often originate either from the patient’s skin or from the surgical team [5, 6]. The most common bacteria causing sternal infections are coagulase negative staphylococci, *Staphylococcus aureus,* and gram-negative bacteria [2, 6–9].

There are different strategies to reduce SSIs in an operating room (OR), such as the use of basic hygiene procedures, controlled OR ventilation, normothermia, surgical techniques, sterile materials, prophylactic antibiotics, and preoperative skin disinfection [10]. A preventive method is to perform preoperative hand disinfection prior to wearing surgical gloves and to double glove for easy detection of puncture in the outer glove [11].

**Fig. 1:**
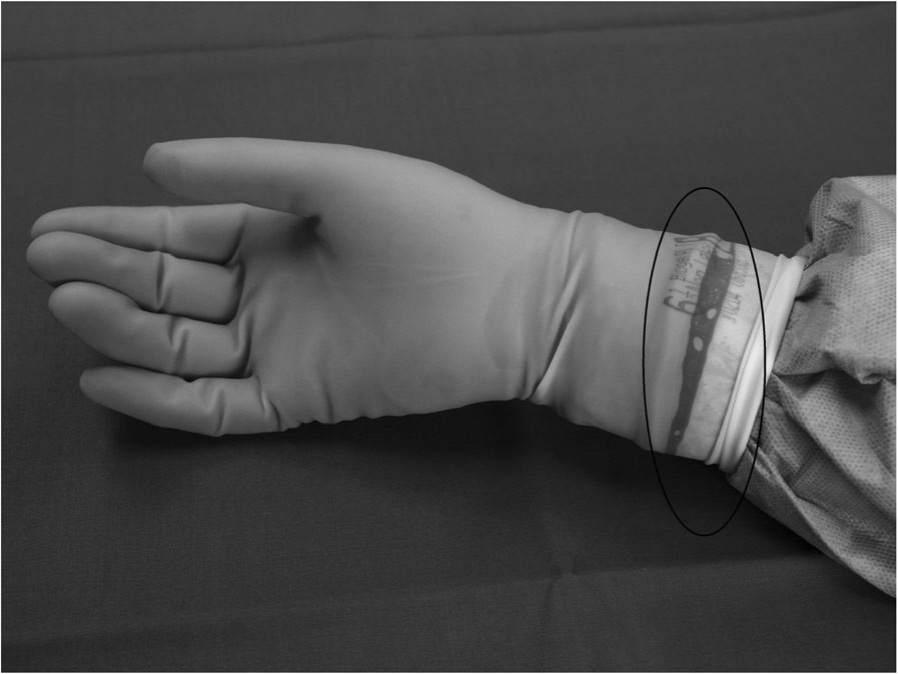
Photo illustrating a dark visible indication of fluid at the glove cuff end

Strategies have been recommended to reduce the incidence of SSIs, and great attention has been focused on the liquid-proof barrier of the surgical gown and gloves [11]. Double gloving has become routine in many departments because of its effect of reducing the risk of transmitting bacteria through puncture of the gloves [11, 12]. Less focus has been centred on the largest hole in the glove, the glove cuff, the place at which the hand enters the glove. There are few studies that address this issue but these studies are not performed recently. However, the issue is nevertheless current because the problem still exists. If, while wearing a liquid-proof surgical gown and surgical gloves, the hands and arms were set under a water tap, the arms would get wet [13, 14]. It has been noted in clinical practice that during surgical procedures, the gloves will become moist at the end of the surgical glove cuff. Often the gloves will turn dark, indicating that fluid is present (Fig. 1), a process that can only be detected by using double gloves with an indicator system by which a darker colour appears. A question to be raised is if the fluid originates from the skin of the hand and if it may contain bacteria.

There may be differences in bacterial growth and recolonization on the hands of OR nurses compared to those of non-health care workers (non-HCWs), due to their frequently performed preoperative hand disinfection and frequent exposure to virulent bacteria [15–19]. A need existed to investigate whether OR nurses had different amounts or type of bacteria present on their hands after preoperative hand disinfection and wearing surgical gloves compared to a control of non-HCWs. No previous study has investigated whether repetitive preoperative hand disinfection affects the results regarding bacterial growth and recolonization on the hands, both directly after the preoperative hand disinfection process and then after wearing surgical gloves.

The aim was to investigate if there were differences in bacterial growth and recolonization of hands between OR nurses and non-HCWs as well as to investigate if bacterial growth existed at the surgical glove cuff and gown interface during surgery.

## Methods

### Study design and participants

This pilot project was designed as an exploratory comparative clinical trial with two groups for comparison. All OR nurses (*n* = 14) employed at one cardiothoracic surgery department in Sweden and non-HCWs (*n* = 14) without any recent contact with medical care were invited to participate. The non-HCWs were adjusted to match the OR nurses, regarding gender. For both groups, exclusion criteria were artificial nails, hand eczema, jewellery or other preoperative hand disinfectant solution than protocol stated. The study has been performed in accordance with Declaration of Helsinki [20] and the Regional Ethical Review Board of Uppsala approved the study (reference number 2013/283). The study participants received oral and written information about the study, and written informed consent was obtained before data collection. The trial was registered at Research web (FOU in Sweden, 117,971) ClinicalTrials.gov (NCT02359708). The data collection for the OR nurses was performed between March 2014 and June 2014 at an OR department in Sweden, and between December 2014 and April 2015 for the healthy participants.

### Procedure for OR nurses

OR nurses, employees at the same OR department, were informed orally of the study and asked to participate in the study at a workplace meeting. Preoperatively, they performed preoperative hand disinfection accordingly to clinic routine by washing their hands under running water with soap and cleaning their nails if necessary for 1 min, and then drying their hands and forearms properly with paper and rubbing hands and forearms with a fluid alcohol (Dax preop 80, CCS Healthcare AB, Sweden). During surgery the OR nurses wore caps, masks, nonwoven surgical gowns (BARRIER, Mölnlycke Heath Care, Gothenburg, Sweden), and double gloves (Biogel PI indicator system, Mölnlycke Health Care, Gothenburg, Sweden). Intraoperative, the OR nurses prepared and assisted at a clean surgery procedure until they were either relieved or the surgery was completed.

### Procedure for non-HCWs

The non-HCWs were recruited from the first author’s circle of friends. The group consisted of office workers and students, all healthy individuals. The trial was performed at three occasions, with a maximum of five persons at a time, and took place at the same OR department as the nurses’ trial. All non-HCWs performed the preoperative hand disinfection with instructions and the assistance of an OR nurse, who also helped them with donning the nonwoven surgical gowns and double gloves (BARRIER® and Biogel PI system®, Mölnlycke Health Care, Gothenburg, Sweden). The gloves used did not contain any bactericidal agency and the size of the gowns and gloves were set by an experienced OR nurse. The non-HCWs performed the preoperative hand disinfection in the same manner as the OR nurses. To simulate nearly the same workload as preparing and assisting a patient for surgery, they performed a heart and lung resuscitation (HLR) course while dressed in gowns, caps, and gloves.

### Data collection procedure

Skin cultures were taken at two time points, directly after the preoperative hand disinfection when the hands were dry, and again after wearing sterile surgical gloves and gowns. The OR nurses were sampled in total at seven sites, and the non-HCWs at six sites. The non-HCWs had six cultures taken because these participants’ gloves and gowns were not kept sterile during the HLR course; the culture from the glove cuff and gown interface was excluded. Sampling was performed by one of the researchers (CW). At the first time point cultures were obtained at three sites on the right hand: (1) in the palm, (2) between the index finger and middle finger, and (3) at the nail/cuticle of the index finger.

Cultures obtained at the second time point, at the end of surgery and the HLR course, respectively, after taking the gloves off, were obtained from (1) the hand palm, (2) between the index finger and the middle finger, and (3) at the nail/cuticle of the index finger (Fig. 1). The OR nurses had an additional culture taken at the glove cuff and gown interface, which was obtained before taking the gloves off.

All cultures were taken using a nylon-flocked swab (ESwab, Copan Italia S.p.A., Brescia, Italy). The swabs were moisturized with two drops of saline and rubbed for 15 s at the skin culture sites. The culture area was approximately 5 mm × 15 mm. At the nail site the area was smaller. The choice of culture swab was chosen for its ability to answer the research question and was based from a study testing its sensibility [21].

Cultures were kept cold until arrival at the Department of Laboratory Medicine, Clinical Microbiology, and analysed according to a specific study protocol. The laboratory technician that performed the analysis was blinded regarding group allocation.

### Culture analysis

The swabs were vortexed, and 50 μL of the media was subcultured on hematin agar medium 4.3% (*w*/*v*) (Columbia Blood Agar Base, Acumedia Neogen Corporation, Lansing, MI, USA) supplemented with 6% (*v*/v) chocolatized defibrinated horse blood and incubated at 36 °C aerobically. Samples were also subcultured on FAA plates (LAB 90 Fastidious Anaerobe Agar 4.6% (w/v); LAB M Ltd., Lancashire, UK), supplemented with 5% (v/v) defibrinated horse blood and incubated under anaerobic conditions (10% H2, 10% CO2, 80% N2) at 37 °C. After 24 and 48 h of aerobic incubation and 5 days of anaerobic incubation, bacterial growth was determined quantitatively (CFU/mL, colony-forming units per mL). Culture diagnostics and species verification were performed based on characteristic colony morphology and using routine diagnostic procedures, including MALDI-TOF mass spectrometry (MicroflexLT and Biotyper 3.1, Bruker Daltonics, Bremen, Germany).

### Statistical analysis

No previous study has been performed on this specific topic so sample size calculation was not possible. This study will enable us to perform a power calculation for future research. Analysis was performed using SPSS version 22 (SPSS Statistics; IBM, Armonk, NY, USA). The Bacterial counts and other non-normal distributed variables were analysed with Mann-Whitney U test and Wilcoxon Sign Ranks test comparing repeated measurements. Categorical variables were evaluated with chi-square test or Fisher’s exact test, as appropriate. Descriptive statistics are presented as means, median, numbers, percentage, confidence interval, standard deviation, and interquartile range (IQR). A *p*-value < 0.05, two tailed, was considered statistically significant.

## Results

**Fig. 2:**
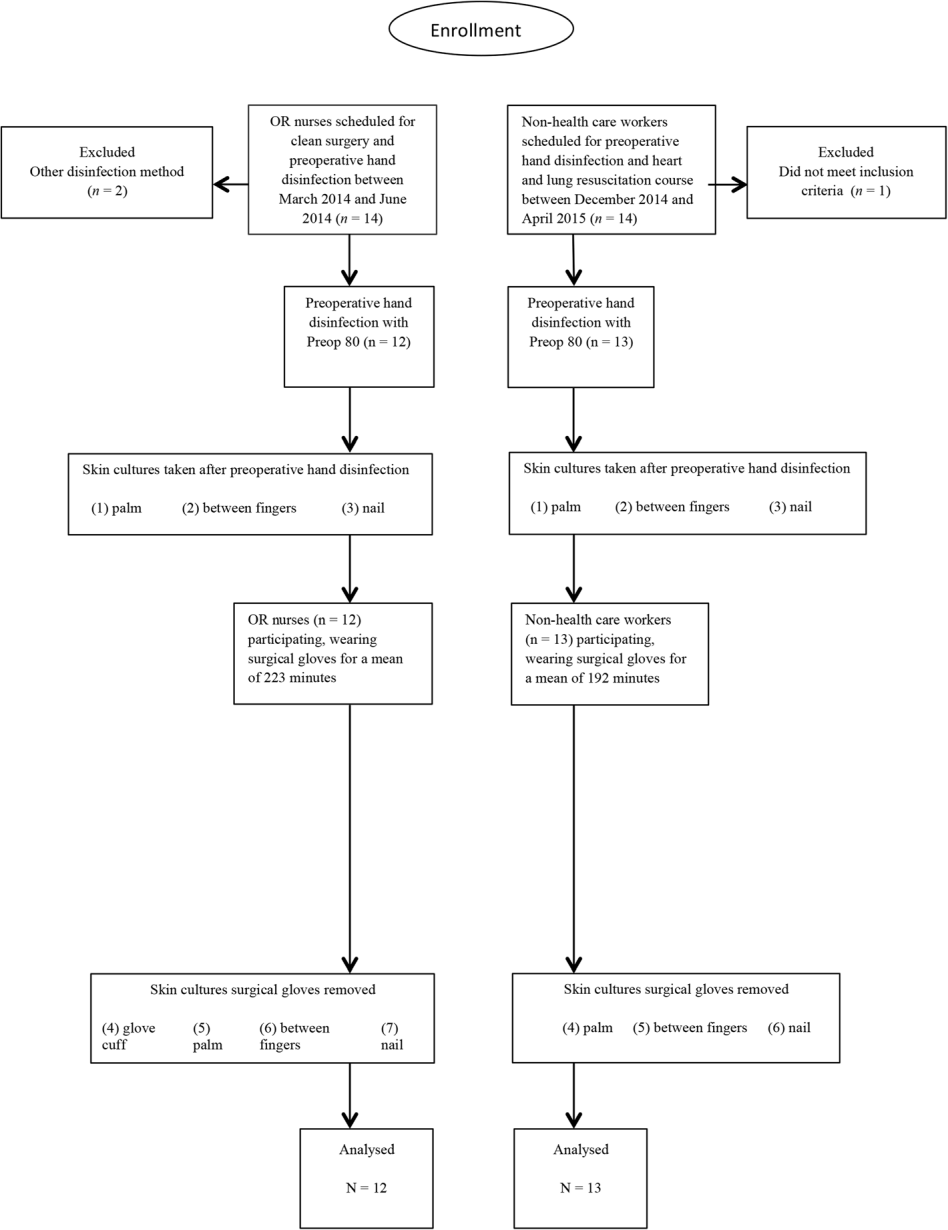
Flow chart of participant inclusion in the study

Two of the 14 OR nurses were excluded due to use of another preoperative hand disinfection method, that is, chlorhexidine containing soap, resulting in a total of 12 OR nurses. One of the 14 non-HCWs was excluded because of nail extensions (Fig. 2), resulting in a total of 13 non-HCW. There were no significant differences between the groups except for the duration of time wearing the surgical gloves (Table 1).

**Table 1:** Comparison of baseline participant characteristics between operating room (OR) nurses and non-health care workers (HCWs)

Characteristic	OR nurses *n* = 12	Non-HCWs *n* = 13	*p*-value
Age, mean (SD)	46 (8)	39 (13)	0.094
Men/women, numbers	1/11	1/12	1.0
Minutes wearing gloves, mean (SD)	223 (34)	192 (16)	0.007^*^

Student’s *t*-test and Fisher’s exact test were used as statistical method

^*^Statistically significant difference

### Bacterial growth and recolonization at the different culture sites

There were differences in bacterial growth and recolonization between the groups at four of six culture sites, regarding the colony-forming units per mL (CFU/mL). After preoperative hand disinfection the OR nurses had higher bacterial growth at the palm and the finger sites compared to the non-HCWs, *p* = 0.044 and *p* = 0.019, but no difference regarding the nail sites *p* = 0.434. After wearing surgical gloves no difference was found regarding the palm site between the groups, *p* = 0.893. OR nurses had higher values regarding recolonization at the finger, *p* = 0.039 but less recolonization at the nail site, *p* = 0.016 compared to non-HCWs (Table 2).

**Table 2:** Bacterial growth and recolonization at three culture sites on the hands of operating room nurses and non-health care workers after preoperative hand disinfection and after wearing surgical gloves

	Culture	Operating room nurses *n* = 12				Non-health care workers *n* = 13				*P*-value
		N (%) growth	CFU/mL mean	CFU/mL median	CFU/mL IQR	N (%) growth	CFU/mL mean	CFU/mL median	CFU/mL IQR	
After hand disinfection	**Palm**	**5 (41.7)**	**13**	**0**	**18**	1 (7.7)	0.8	0	0	**0.044** ^*^
	**Finger**	**6 (50.0)**	**14**	**5**	**18**	1 (7.7)	0.8	0	0	**0.019** ^*^
	Nail	8 (66.7)	28	10	65	5 (38.5)	52	0	40	0.434
After wearing gloves	Palm	4 (33.3)	18	0	8	3 (23.1)	29	0	5	0.893
	**Finger**	**4 (33.3)**	**98**	**0**	**10**	0	0	0	5	**0.039** ^*^
	**Nail**	10 (83.3)	244	55	463	**12 (92.3)**	**1554**	**780**	**2940**	**0.016** ^*^

Mann-Whitney U Test was used to calculate the numbers of bacterial differences at the different culture sites in CFU/mL between the groups

*IQR* interquartile range

^*^Statistically significant difference. Entries in bold face represent significantly more bacterial growth and its relationship with the significance in *P*-value

### Total CFU/mL after preoperative hand disinfection and after wearing surgical gloves

There were no differences between the groups in total CFU/mL, that is, all sampling sites. After preoperative hand disinfection the OR nurses had a median bacterial growth of 35 (IQR 88) versus non-HCWs, who had 0 (IQR 40), *p* = 0.186. The difference in CFU/mL between the OR nurses and the non-HCWs after wearing surgical gloves was 105 (IQR 453) vs. 790 (IQR 2970), *p* = 0.06.

### Differences within the groups

The median of CFU/mL, that is, the three sites all together, on the hands within the OR nurses after preoperative hand disinfection showed a median of 35 compared to 105 CFU/mL after wearing gloves, *p* = 0.031. Within the non-HCWs the median of CFU/mL was 0 vs. 790 CFU/mL, *p* = 0.002.

### Number of persons with growth and recolonization

The number of OR nurses who had growth at any sampling site after preoperative hand disinfection, before donning gloves, was 10 out of 12 (83%), compared to 6 out of 13 (46%) for the non-HCWs, *p* = 0.053. The number of OR nurses who had bacterial growth after wearing gloves was 11 out of 12, while all of the non-HCWs had growth in one or more of the three culture sites.

### Isolated bacteria

Fourteen different bacterial species were found. The most frequent species obtained from the OR nurses were *Staphylococcus warneri* followed by *Propionibacterium acnes* and in the non-HCWs group *S. warneri* followed by *Staphylococcus epidermidis* together with *Staphylococcus pasteuri* (Table 3).

**Table 3:** Bacterial species isolated from the hands of operating room (OR) nurses and non-health care workers (HCWs). Numbers of persons with specific bacteria after preoperative hand disinfection and after wearing sterile gloves

	Bacterial growth after preoperative hand disinfection		Bacterial recolonization after wearing sterile gloves	
	OR nurses	Non-HCWs	OR nurses	Non-HCWs
	*n* = 12	*n* = 13	*n* = 12	*n* = 13
^*^Minutes, mean (SD)			223 (34)	192 (16)
Persons with growth, (%)	10 (83)	6 (46)	11 (92)	13 (100)
*S. warneri*	4	2	6	11
*P. acnes*	4	1	5	3
*Bacillus* sp.	5	1	4	4
*S. epidermidis*	–	2	5	4
*S. capitis*	3	1	3	–
*S. pasteuri*	–	2	–	4
*Micrococcus* sp.	3	–	3	–
*S. haemolyticus*	1	–	1	1
Alpha-haemolytic streptococci	1	–	2	1
*Brevibacteriaceae*	–	1	–	1
*S. lugdunensis*	–	1	–	1
*Gemella haemolysans*	–	–	1	–
Gram-positive cocci, non-typeable	–	–	–	1
*Enterobacteriaceae*	–	1	–	–

^*^Significant difference between groups regarding minutes, *p* = 0.007; statistical method used was student’s *t*-test

### Bacteria present during surgery

Nine out of 12 (75%) of the OR nurses had a visible dark area around the glove cuff and gown interface, indicating fluid, and in five of them (42%) there was bacterial growth. Four of five cultures from the OR nurses’ had the same bacteria at the glove cuff and gown interface as found in the cultures from the hands. Bacteria isolated from the cultures were *P. acnes*, *S. warneri*, *S. epidermidis* and *Micrococcus* species. The CFU/mL ranged from 10 to 40 at the interface of the surgical glove cuff and gown.

## Discussion

This pilot study was conducted at one specific OR department and the group of non-HCW was the first author’s circle of friends. The selection of non-HCW’s focused on people not having contact with hospital environment and maintaining ordinary hand hygiene. Both groups were either work friends or personal friends which should not affect the bacterial flora of the participants. Nor was it suitable for the non-HCW’s to participate during surgical procedures, hence the design with different tasks for each group. The aim of preoperative hand disinfection is to eradicate transient flora and reduce resident flora of the hands and promote a prolonged effect [22]. The preoperative hand disinfection solution used in this study was approved according to EN Standard EN12791. The glove juice technique recommended in a Cochrane review was not applicable for this specific sample site [24]. The technique used in our present study was chosen to investigate if bacterial growth existed at the surgical glove cuff and gown interface. This means that the culture had to be sampled at the glove cuff and gown interface. It have been reported that recolonization occurs inside the gloves and that the bacterial counts on the hands increases with time [23, 24], suggesting that the skin disinfection reduce bacteria on the skin but that bacteria persists deeper down in the skin pores and hair follicles after the perioperative hand disinfection process. The amount of bacterial growth found at the glove cuff end were not very high compared to the amount of bacterial growth sometimes found on the hands after wearing gloves but this shows that the glove cuff end is a danger zone which can transfer bacterial growth, possibly from the hands, but the glove cuff may also be contaminated by the hands when donning the glove [25, 26]. In the present study the amount of bacteria was greater in OR nurses than in non-HCWs at the beginning of wearing gloves, whereas the opposite occurred after wearing surgical gloves. It seems as the recolonization rate was higher in the group consisting of non-HCWs. The duration of wearing gloves may have an impact on the recolonization rate [23]. Moreover, damaged skin is more likely to have a higher amount of bacteria compared to healthy skin [27]. The risk that more of the OR nurses had damaged skin than the non-HCWs are probable higher due to the extensive hand wash regime OR nurses perform every day at work. Considering that the OR nurses wore the surgical gloves for a significantly longer period of time and had a greater amount of bacteria on their hands at the start, they still had a lower bacterial recolonization compared to the non-HCWs. This may suggest that repeatedly performing hand disinfection inhibits the bacterial recolonization rate of the hands, but this has to be further investigated.

This pilot study was performed to investigate whether bacterial growth from the surgical glove cuff and gown interface could be found, since bacterial growth was suspected, and no previous study had addressed this issue. The bacteria present at the surgical glove cuff and gown interface were the types that could cause SSIs [6]. It is speculated that bacteria might migrate from the skin of the hands to the surgical glove cuff and further on to the sleeve of the sterile surgical gown. It is unlikely that bacteria will pass through the material of the gown when the gown used is liquid proof. The origin of the fluid is most likely to be sweat or evaporations from the user’s hands, but this was not tested in present study. It is also noted in clinical practice that the sleeves of the surgical gloves roll down and turn inside out. In some OR departments’ staff routinely seal the inner gloves with sterile sticky tape to prevent the insides of the gloves being exposed. Our study has indicated that bacterial growth seems to exist between the surgical glove cuff and gown interface. In an attempt to enhance the barrier, some clinicians put on the inner glove before the gown. When the sterile gown is on, they use sterile scissors to make a small hole in the gown cuff so that the thumb can pass through. When this is done the outer glove can be donned. This technique can be seen in a technical note by Fernandez and colleagues; note, however, that they use this technique for another reason and use only single gloves [28]. Sealing the inner glove and changing the order of gloves and gowns might prevent bacterial leakage; this has still to be investigated.

There is a need to develop surgical gloves with a secure interface between gloves and gowns. A suggestion is to develop a one-piece gown with sealed inner gloves attached directly to the gown. Gown size could follow the glove size. The limitation of a one-piece garment incorporating gown and gloves would be the capability of changing only outer gloves. Yet, a change of inner gloves is seldom needed [29].

### Limitations

A limitation in the design was the lack of bacterial baseline for the two groups. It is possible that the groups had significantly different amount of bacteria at the start of the preoperative hand disinfection procedure and this may have affected the results. Furthermore, the differences in performed activities between the two groups during data collection i.e. surgery vs HLR, may have influenced the results. As well as the lack of experience in the non-HCWs group how to perform the preoperative hand disinfection and donning gowns and gloves, even though they were assessed by an OR nurse.

The reliability of taking cultures can be questioned and no method is yet perfect. One person performed all the skin samples according to a pre-set way which minimize bias regarding possible differences in sampling technique. At the department of clinical microbiology, a study specific protocol was prepared for the sample analysis to strengthen the method.

## Conclusions

Although the outcome data may be taken by caution, this pilot study showed bacterial growth existed at the glove cuff end, and that it mostly was the same bacteria as found on the hands which indicates a need for more secure surgical glove and gown interface to avoid cross contamination during surgery. It seems like OR nurses have a more difficult task eradicating bacterial growth with preoperative hand disinfection but on the other hand they have less bacterial recolonization rate compared to non-HCW. Both groups had significantly larger amount of bacterial growth after wearing sterile gloves compared to directly after the preoperative hand disinfection indicating rapid recolonization.

## Abbreviations

CFU/mL: Colony-forming units per Ml
HLR: Heart and lung resuscitation
IQR: Inter quartile range
*n*: *Number*
Non-HCW: Non-health care workers
OR: Operating room
*p*: *p-*value
SSI: Surgical site infection
